# A Novel Combined Use of Dupilumab for Treatment of Aggressive Refractory Pemphigus Vulgaris Complicated With Pulmonary Tuberculosis: A Case Report and the RNA-seq Analysis

**DOI:** 10.3389/fimmu.2022.825796

**Published:** 2022-02-09

**Authors:** Siji Chen, Shaowei Zhan, Chunting Hua, Yi Tang, Hao Cheng

**Affiliations:** ^1^ Department of Dermatology and Venereology, Sir Run Run Shaw Hospital, Zhejiang University School of Medicine, Hangzhou, China; ^2^ Department of Dermatology, Zhejiang Provincial People’s Hospital, People’s Hospital of Hangzhou Medical College, Hangzhou, China

**Keywords:** pemphigus vulgaris (PV), dupilumab, autoimmune disease, RNAseq analysis, biologic agents (biologies), case report

## Abstract

**Background:**

Pemphigus vulgaris (PV) is a kind of IgG-mediated autoimmune blistering disease (AIBD) that is characterized by loss of keratinocyte adhesion in the epithelium of mucous membranes or skin. Recently, pemphigus vulgaris was thought to be associated with classical T helper 2 (T_H2_)-type cytokines such as interleukin‐4 (IL-4) and interleukin‐17 (IL-17) signaling pathway. A humanized monoclonal IgG4 antibody called dupilumab binds to the alpha subunit of the interleukin‐4 receptor (IL‐4Rα) and inhibits the signaling of IL-4 and interleukin‐13 (IL-13), which has been successfully applied for atopic dermatitis and asthma. Currently, the clinical trial evaluating dupilumab in bullous pemphigoid is ongoing.

**Objective:**

To determine whether dupilumab may be of benefit in the aggressive refractory pemphigus vulgaris.

**Methods:**

We report a 35-year old male with refractory pemphigus vulgaris and pulmonary tuberculosis who received treatment with dupilumab for 10 weeks. The mRNA expression of peripheral blood mononuclear cells (PBMCs) was analyzed by RNA sequencing (RNA-seq) which showed the gene expression changes after treatment.

**Results:**

The skin lesions of the patient improved in response to the combined use of dupilumab, moderate dose of glucocorticosteroids, and intravenous immune globulin (IVIG). Downregulations of inflammatory response-related genes and IL-17 signaling pathway-related genes were observed in PBMCs.

**Conclusion:**

We describe a patient with refractory pemphigus vulgaris and pulmonary tuberculosis who had the disease under control with combined use of dupilumab as an add-on treatment. Dupilumab may provide a beneficial effect in aggressive refractory pemphigus vulgaris.

## Background

**Figure 1:**
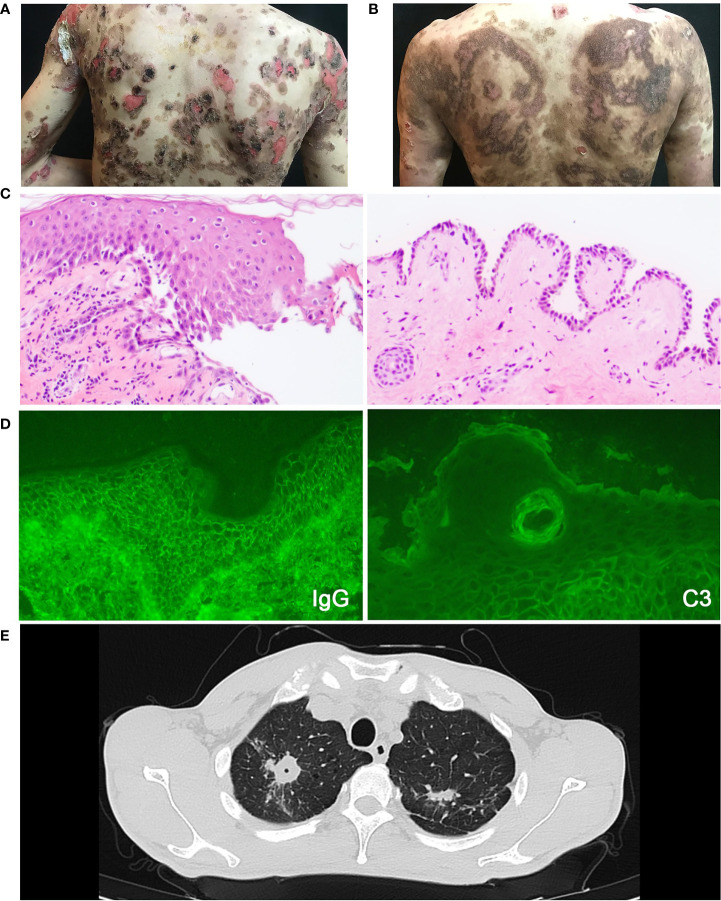
The clinical photographs of patient, essential elements for pemphigus and pulmonary tuberculosis diagnosis. **(A)** The clinical photograph taken on admission to the hospital. **(B)** The clinical photograph taken on one-week follow-up. **(C)** Histopathological image of the lesions, which shows intraepithelial cleavage with detached keratinocytes primarily localized to the suprabasal region. **(D)** Intercellular deposition of IgG and C3 by direct immunofluorescence microscopy. **(E)** The chest CT scan of pemphigus patient with pulmonary tuberculosis showing lesions in bilateral upper lungs.

Pemphigus vulgaris is a severe autoimmune blistering disease (AIBD) affecting mucous membranes and the epidermis with autoantibodies mainly targeting cell-to-cell adhesion molecules desmoglein 3 (Dsg3) and desmoglein 1 (Dsg1) (1). The most commonly used treatment is systemic glucocorticosteroids combined with or without adjuvant nonsteroidal immunomodulatory agents such as azathioprine and mycophenolate mofetil. Refractory pemphigus vulgaris is generally referring to pemphigus vulgaris that fails to respond sufficiently to an optimal dose of first-line treatment. The treatment potions of refractory pemphigus vulgaris include glucocorticosteroids plus Rituximab or high-dose intravenous immune globulin (IVIG). Despite the introduction of biologic agents in recent years, the treatment of refractory pemphigus vulgaris especially pemphigus vulgaris with serious complications remains challenging. For many years, the T helper 2 (T_H2_)-related cytokines such as interleukin‐4 (IL-4) and interleukin‐17 (IL-17) signaling pathway are gaining attention to the pathogenesis and treatment of pemphigus vulgaris (2–4). In addition, investigators have found Dsg-reactive IL-4^+^ T_H2_ cells in the peripheral blood of pemphigus patients. Dupilumab, a humanized monoclonal IgG4 antibody binding to the alpha subunit of the interleukin‐4 receptor (IL‐4Rα), has already been applied for atopic dermatitis and asthma. A multi-institutional study in 2020 showed that bullous pemphigoid patients who received dupilumab achieved a well-tolerated satisfactory control of the disease, but may need higher doses than atopic dermatitis (5). In the past five years, a series of case reports have described the use of dupilumab in the treatment of bullous pemphigoid (6). There is an ongoing recruiting, multicenter, randomized, double-blind, placebo-controlled, and parallel-group clinical trial to evaluate the efficacy and safety of Dupilumab in adult bullous pemphigoid patients (NCT04206553). Here, we combine the clinical features and RNA-seq analysis to describe a patient with refractory and aggressive pemphigus vulgaris with pulmonary tuberculosis (TB) whose pemphigus-related symptoms were successfully improved with a combination treatment of dupilumab (**Figures 1A, B**).

## Case Presentation

A 35-year-old male had a half-year history of blistering and ulceration from the oral mucosa to the trunk, limbs, glans, genital skin, and mucosa with pruritus and scabs, showing no response to antibiotic therapy. Upon physical examination, several flaccid blisters and superficial skin erosions involving the oral mucosa were seen in more than 70% of the body surface area, with the Nikolsky’s signs (direct and indirect) positive. The diagnosis of pemphigus vulgaris was made based on the presence of typical clinical features, consistent histopathological findings (intraepithelial cleavage with detached keratinocytes primarily localized to the suprabasal region) (**Figure 1C**), and positive direct immunofluorescence assessments (spinosum intercellular deposition of immunoglobulin G and complement component 3) (**Figure 1D**). There was a high blood eosinophil count (1.04 × 10^9^/L, normal range 0.02–0.52 × 10^9^/L) and a high serum IgE level (374 IU/ml; normal range <165 IU/ml).

**Figure 2:**
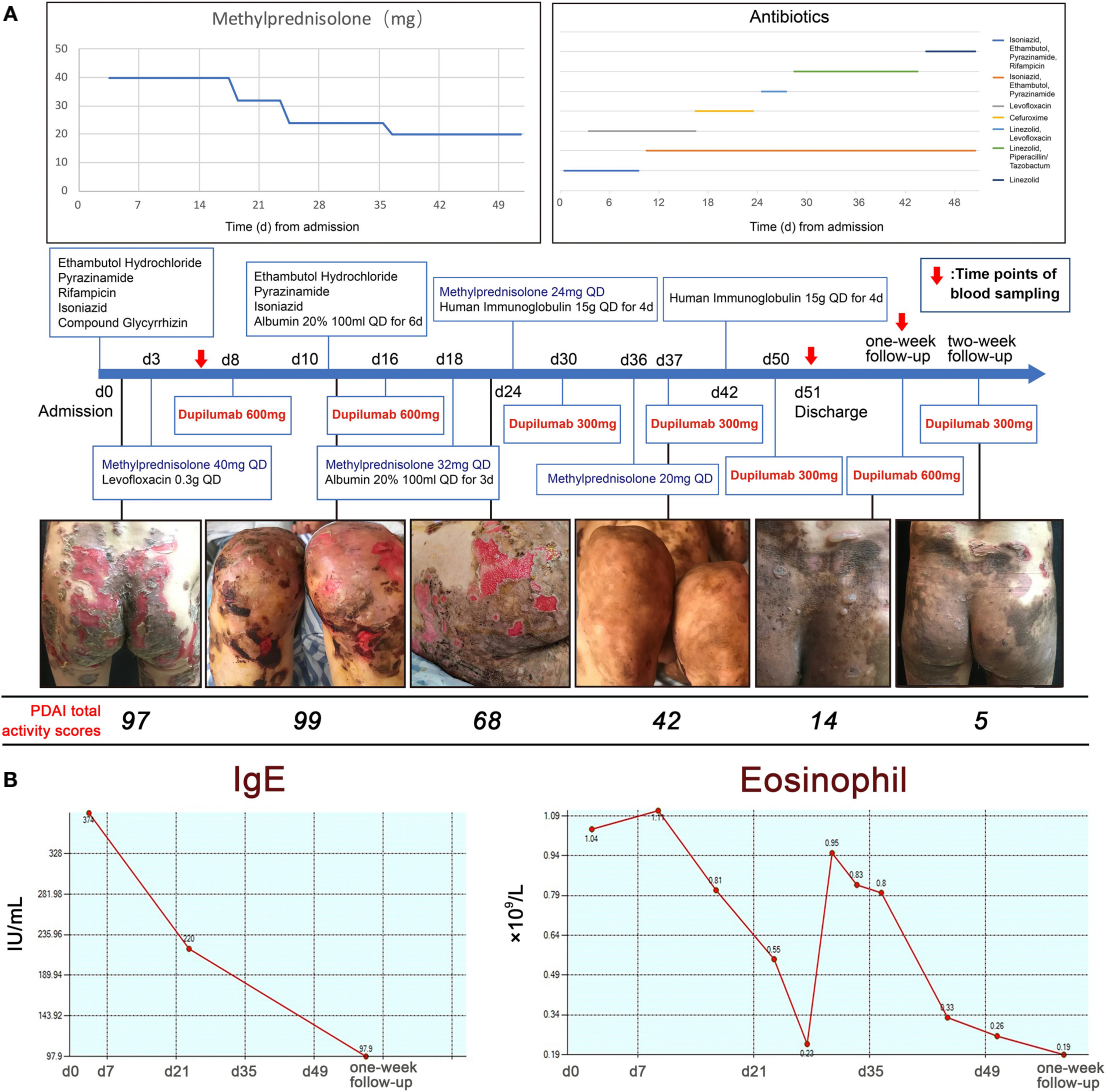
The use of medications and the trends in blood IgE and eosinophil counts. **(A)** The use of medications including methylprednisolone, antibiotics and dupilumab with clinical photographs and PDAI total activity scores. **(B)** IgE levels and eosinophil counts during hospitalization.

However, the patient had low fever and night sweats at the time of the first hospitalization. The chest CT scan showed lesions located in the bilateral upper lungs and the right lower lung which were considered as TB (**Figure 1E**). Then the patient was discharged and treated with Anti-tuberculous chemotherapy (daily rifampin 75 mg, isoniazid 37.5 mg, pyrazinamide 200 mg, and ethambutol 137.5 mg) without pemphigus vulgaris treatment in the designated hospital for TB. The patient was readmitted to our hospital 2 months later due to increasing blister erosions and ulcers throughout the body. He firstly received a moderate dose of glucocorticoids (methylprednisolone 40 mg/day) with poor efficacy, and the condition progressed rapidly (**Figure 2A**). The patient showed a sleep disturbance (trouble falling asleep and sleep-talking), slightly obtunded mental status with fever, and blood inflammatory-related indicators increased (white cell counts, ESR, and hypersensitive C-reactive protein). ESBL-positive *Escherichia coli* and *K. pneumoniae* were found in the wound culture. The psychiatric, neurology and infectious disease section consultations considered bacterial or tuberculous intracranial infection or sepsis may exist. Based on the conditions above, the dose of methylprednisolone could not be further increased. It is also difficult to use rituximab which may induce a prolonged B-cell depletion and aggravate infections (7). To improve therapy, dupilumab (600 mg subcutaneously initially and 300 mg subcutaneously every other week) was added to his regimen combined with methylprednisolone, anti-TB regular regimen, and antibiotics plus low-dose intravenous immunoglobulin (15 g IVIG for 4 d) for anti-infection (**Figure 2A**).

Under this combination therapy, the symptoms improved within one and a half months, while few new inflammatory lesions developed and pruritus relief. Pemphigus disease area index (PDAI) total activity scores decreased from 97 to 42 (**Figure 2A**) (8). At the same time, both blood IgE level and eosinophils counts decreased, which may elevate in acute onset pemphigus vulgaris patients (9) (**Figure 2B**). The patient was discharged with prolonged maintenance treatment with low-dose methylprednisolone and dupilumab. New onset of blistering was observed on the hips of the patient at first-week follow-up (PDAI total activity score = 14). The new skin lesions regressed rapidly (PDAI total activity score = 6) in one week after using dupilumab (600 mg subcutaneously).

## RNA-Sequence Analysis

**Figure 3:**
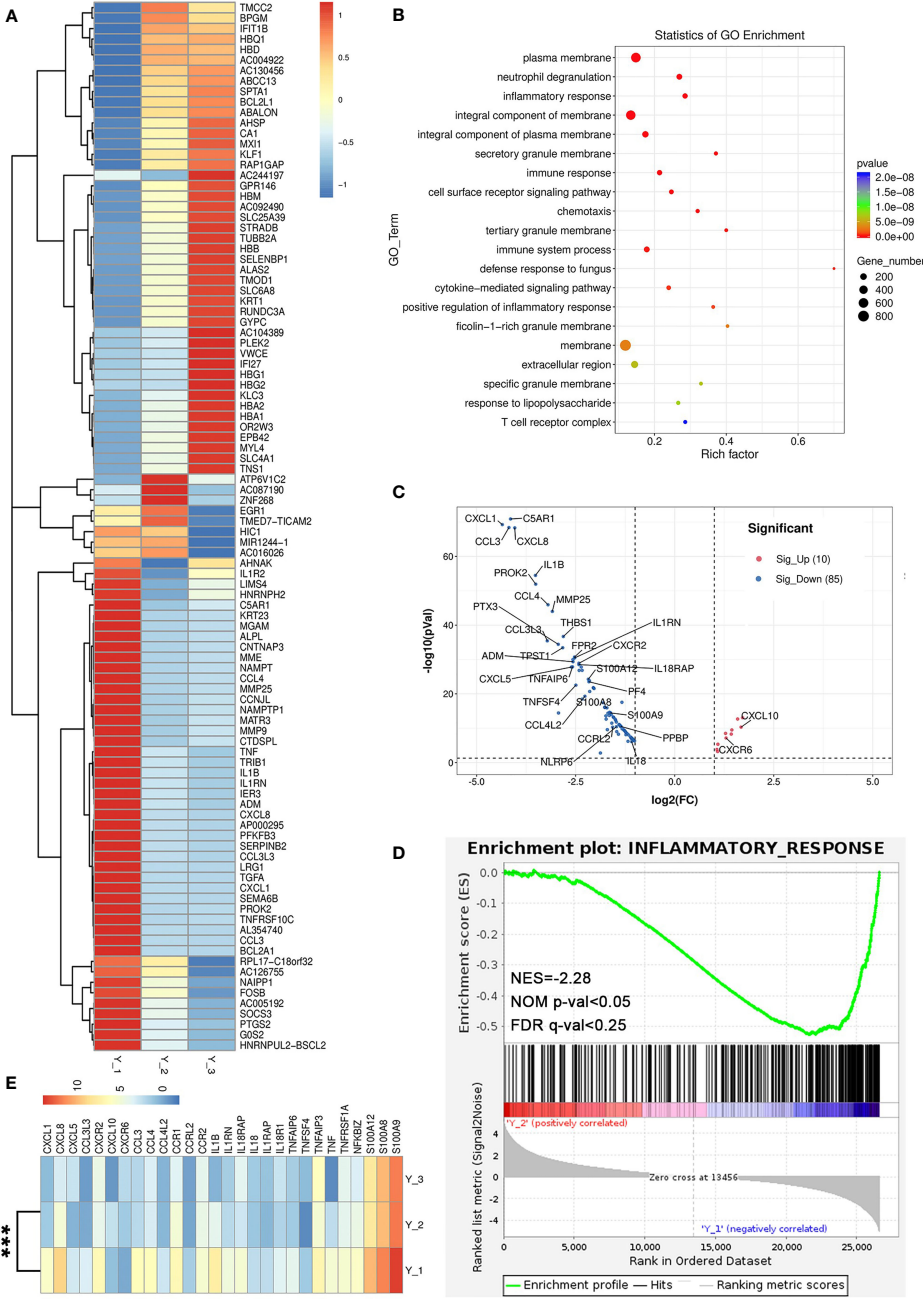
The analysis of RNA-seq. **(A)** The differential genes of three time points. **(B)** Dot plot of GO enrichment analysis of differential genes between Y_2 and Y_1. **(C)** Volcano plot showing the upregulated genes (red, 10) and downregulated genes (blue, 85) of inflammation response-related genes. **(D)** The GSEA enrichment analysis of the inflammatory response-related genes between Y_2 and Y_1. **(E)** Heatmap shows the differential genes of inflammatory response-related genes (***p < 0.01).

Currently, there is still no RNA sequence about peripheral blood immune cells of pemphigus vulgaris. After signing the informed consent, the blood of the patient was obtained from three time points: hospitalization (Y_1), discharge (Y_2), and one-week follow-up (Y_3). RNA-seq was performed after extraction of PBMCs using TRlzol to obtain differential genes in the three states of the patient (**Figure 3A**). We constructed a cDNA library by technology from the pooled RNA from PBMC samples which were sequenced run with Illumina Novaseq™ 6000 sequence platform and then aligned reads of all samples to the human reference genome using HISAT2 package (https://daehwankimlab.github.io/hisat2/, version: hisat2-2.0.4) (10). The Gene Ontology (GO) enrichment analysis (http://www.geneontology.org/) between Y_2 and Y_1 showed that a total of 275 GO entries were obtained and top20 (p <0.01) were selected (**Figure 3B**), namely, plasma membrane (GO: 0005886), neutrophil degranulation (GO: 0043312), inflammatory response (GO: 0006954), and immune response (GO:0006955). According to inflammatory response, there were upregulation of 10 genes and downregulation of 85 genes in the PBMCs of the patients when discharged (**Figure 3C**). A Gene Set Enrichment Analysis (GSEA, software.broadinstitute.org/gsea/index.jsp) was performed for GO terms, where the downregulation of inflammatory response was significant (NES = −2.2, NOM p-value <0.05, FDR q-value <0.25) (**Figure 3D**). We also investigated the differentially expressed genes about C–X–C motif chemokine ligand chemokine family (CXCL1, CXCL5), interleukin (IL1B, IL1RAP, IL18), C–C motif chemokine ligand chemokine family (CCL3, CCL4), and inflammatory cytokines (S100A8, S100A9, S100A12, TNF) were significantly decreased (p <0.01) (**Figure 3E**).

**Figure 4:**
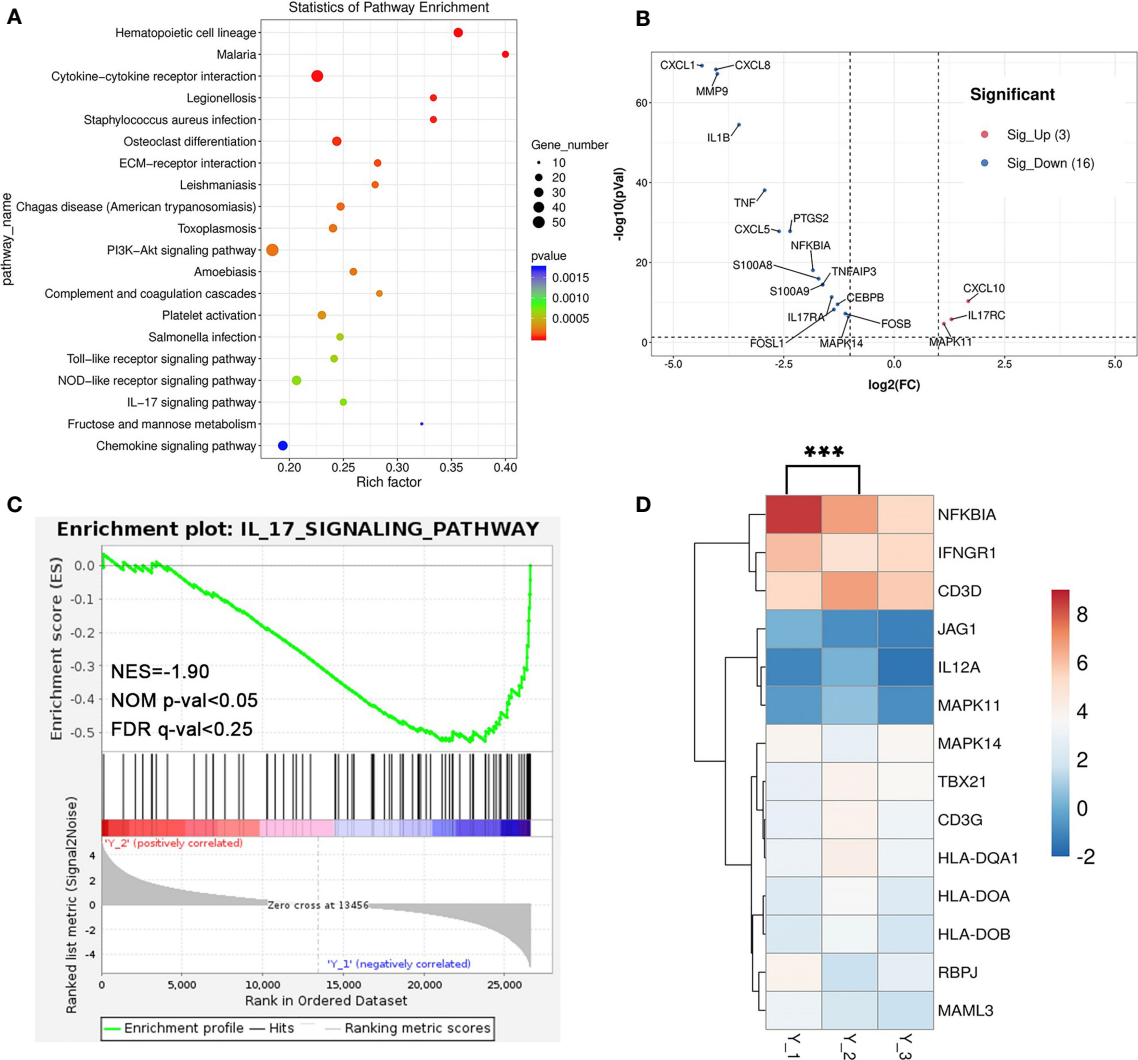
The analysis of RNA-seq. **(A)** Dot plot of KEGG enrichment analysis of differential genes between Y_2 and Y_1. **(B)** Volcano plot showing the upregulated genes (red, 3) and downregulated genes (blue, 16) of IL-17 signaling pathway-related genes. **(C)** The GSEA enrichment analysis of the IL-17 signaling pathway-related genes between Y_2 and Y_1. **(D)** Heatmap shows the differential genes of T_H1_ and T_H2_ cell differentiation-related genes (***p < 0.01).

Interleukins such as IL-4 and IL-17 are thought to play a key role in pemphigus, where high frequencies of circulating IL-17-producing T-cell subsets could be found in active pemphigus vulgaris patients’ peripheral blood, and T_H17_ cells correlate with the levels of Dsg3-specific B cells (1). The Kyoto Encyclopedia of Genes and Genomes (KEGG) database (http://www.genome.jp/kegg/) between Y_2 and Y_1 showed that a total of 32 KEGG pathway enrichment entries were obtained and the top 20 (p <0.01) were selected (**Figure 4A**), including cytokine–cytokine receptor interaction (ko04060), PI3K-Akt signaling pathway (ko04151), and IL-17 signaling pathway (ko04657). The expression of the IL-17 signaling pathway-related genes such as CXCL1, CXCL8, IL1B, IL17RA, and FOSA decreased after treatment (**Figure 4B**). GSEA was performed for KEGG pathways, where the downregulation of IL-17 signaling pathway was significant (NES = −1.90, NOM p-value <0.05, FDR q-value <0.25) (**Figure 4C**). We also found that the expression of T_H1_ and T_H2_ cell differentiation-related genes such as NFKBIA and JAG1 decreased (**Figure 4D**). These findings showed that after the combined use of dupilumab, the levels of mRNA of inflammatory response and IL-17 signaling pathway downregulated, while some genes of T_H1_ and T_H2_ cell differentiation-related genes expression in patients also changed. This finding may reflect the remission of the pemphigus vulgaris after treatment.

## Discussion

Treatment of pemphigus vulgaris is still challenging, especially when systemic glucocorticosteroids and nonsteroidal immunomodulatory agents are relatively contraindicated. Biologic agents can be used as a safer and more efficient choice of treatment in such patients. For many years, T_H2_ cells were thought to be important to form IgG1 and IgG4 autoantibodies against Dsgs (4, 11). Furthermore, IgE-related signaling *via* IL-4 on T_H2_ cells is a novel target of pemphigus pathology (9, 12), so dupilumab may be a viable choice of treatment for pemphigus vulgaris. Here, we present the concomitant use of dupilumab, the IL-4 blocker, and the standard therapy in a patient with refractory pemphigus vulgaris and pulmonary tuberculosis for the first time, after the previous therapies adopted with a moderate dose of glucocorticoids had failed. Because of the infection with tuberculosis and suspicions of intracranial infection or sepsis, higher dosages of systemic glucocorticosteroids and rituximab were unfavorable for this patient. After the use of dupilumab, patients showed a significant decrease in eosinophil and IgE counts while 80% of erosions healed with skin pigmentation after 10 weeks. Drops of serum IgE level and eosinophil counts are also thought to be a response to dupilumab, which blocks the intercellular signaling of IL-4 and IL-13, cytokines that help maintain the immune response of T_H2_ cells.

Dupilumab highlights a specific clinically important T_H2_ pathway in the successful control of pruritus, which is important in atopic dermatitis and autoimmune disease. Dupilumab targets these disease-driving mechanisms inhibiting IL-4 and IL-13 signaling and indirectly downregulates IgE secretion and eosinophils activity as well. It is also thought to reduce signals of peripheral itch sensory. We suggested a higher dose of dupilumab for pemphigus vulgaris compared with application in atopic dermatitis. Our RNA-seq showed a decrease in inflammatory-related genes, IL-17 signaling pathway-related genes, and IL-4-related receptors in PBMCs of patients after treatment, demonstrating the role of dupilumab in the treatment of aggressive refractory pemphigus vulgaris with TB might be extremely valuable.

The patient was grateful and willing to continue using dupilumab, although he found the dupilumab is costly as it is not covered by his health insurance. Dupilumab has been reported to control atopic dermatitis with administration starting at a dose of 600 mg subcutaneously, followed by 300 mg every two weeks. In analogous with eosinophilic esophagitis and bullous pemphigoid, weekly dosing may be necessary for patients with refractory pemphigus vulgaris (5). Therefore, we hypothesized that the downstream mechanisms of action in pemphigus vulgaris are involved in dupilumab treatment.

Several limitations are noted, namely, the small sample size and the short follow-up period. The circulating pemphigus autoantibodies value was not available because the poor condition of the patient could not allow him to go to another hospital where this test could be performed. The RNA-seq could just reflect a clinical situation. Present data is insufficient to demonstrate dupilumab could be effective in other pemphigus subsets. Further experimental and clinical studies are still needed to assess the viability and efficacy of dupilumab for the treatment of pemphigus vulgaris.

## Data Availability Statement

The original contributions presented in the study are publicly available. This data can be found here: https://www.ncbi.nlm.nih.gov/geo/query/acc.cgi?acc=GSE190024.

## Ethics Statement

The studies involving human participants were reviewed and approved by the Sir Run-Run Shaw Hospital Ethical Committee. The patients/participants provided their written informed consent to participate in this study. Written informed consent was obtained from the individual(s) for the publication of any potentially identifiable images or data included in this article.

## Author Contributions

SC and HC designed the manuscript and figures. SZ designed the manuscript. CH and YT revised the manuscript. HC supervised the study, revised the final version of the manuscript and responsible for supervision and funding acquisition. All authors listed have made a substantial, direct, and intellectual contribution to the work and approved it for publication.

## Funding

This work was supported by grants from the Natural Science Foundation of Zhejiang Province (2018C04013) and the Foundation from the Health Bureau of Zhejiang Province (2019PY037).

## Conflict of Interest

The authors declare that the research was conducted in the absence of any commercial or financial relationships that could be construed as a potential conflict of interest.

## Publisher’s Note

All claims expressed in this article are solely those of the authors and do not necessarily represent those of their affiliated organizations, or those of the publisher, the editors and the reviewers. Any product that may be evaluated in this article, or claim that may be made by its manufacturer, is not guaranteed or endorsed by the publisher.

## References

[1] Kasperkiewicz M Ellebrecht CT Takahashi H Yamagami J Zillikens D Payne AS. Pemphigus. Nat Rev Dis Primers (2017) 3:17026. doi: 10.1038/nrdp.2017.26 28492232 PMC5901732

[2] Holstein J Solimani F Baum C Meier K Pollmann R Didona D. Immunophenotyping in Pemphigus Reveals a TH17/TFH17 Cell–Dominated Immune Response Promoting Desmoglein1/3-Specific Autoantibody Production. J Allergy Clin Immun (2021) 147:2358–69. doi: 10.1016/j.jaci.2020.11.008 33221382

[3] Veldman C Stauber A Wassmuth R Uter W Schuler G Hertl M. Dichotomy of Autoreactive Th1 and Th2 Cell Responses to Desmoglein 3 in Patients With Pemphigus Vulgaris (PV) and Healthy Carriers of PV-Associated HLA Class II Alleles. J Immunol (Baltimore Md 1950) (2003) 170:635–42. doi: 10.4049/jimmunol.170.1.635 12496453

[4] Gudjonsson JE Kabashima K Eyerich K. Mechanisms of Skin Autoimmunity: Cellular and Soluble Immune Components of the Skin. J Allergy Clin Immun (2020) 146:8–16. doi: 10.1016/j.jaci.2020.05.009 32631499

[5] Abdat R Waldman RA de Bedout V Czernik A Mcleod M King B. Dupilumab as a Novel Therapy for Bullous Pemphigoid: A Multicenter Case Series. J Am Acad Dermatol (2020) 83:46–52. doi: 10.1016/j.jaad.2020.01.089 32179082

[6] Russo R Cozzani E Gasparini G Parodi A. Targeting Interleukin 4 Receptor α: A New Approach to the Treatment of Cutaneous Autoimmune Bullous Diseases? Dermatol Ther (2020) 33:e13190. doi: 10.1111/dth.13190 31863534 PMC7154653

[7] Sinagra JL Vedovelli C Binazzi R Salemme A Moro F Mazzanti C. Case Report: Complete and Fast Recovery From Severe COVID-19 in a Pemphigus Patient Treated With Rituximab. Front Immunol (2021) 12:665522. doi: 10.3389/fimmu.2021.665522 33936104 PMC8087171

[8] Rosenbach M Murrell DF Bystryn J Dulay S Dick S Fakharzadeh S. Reliability and Convergent Validity of Two Outcome Instruments for Pemphigus. J Invest Dermatol (2009) 129:2404–10. doi: 10.1038/jid.2009.72 PMC3010359 19357707

[9] Nagel A Lang A Engel D Podstawa E Hunzelmann N de Pita O. Clinical Activity of Pemphigus Vulgaris Relates to IgE Autoantibodies Against Desmoglein 3. Clin Immunol (Orlando Fla) (2010) 134:320–30. doi: 10.1016/j.clim.2009.11.006 20015693

[10] Kim D Langmead B Salzberg SL. HISAT: A Fast Spliced Aligner With Low Memory Requirements. Nat Methods (2015) 12:357–60. doi: 10.1038/nmeth.3317 PMC4655817 25751142

[11] Takahashi H Kuwana M Amagai M. A Single Helper T Cell Clone Is Sufficient to Commit Polyclonal Naive B Cells to Produce Pathogenic IgG in Experimental Pemphigus Vulgaris. J Immunol (Baltimore Md 1950) (2009) 182:1740–5. doi: 10.4049/jimmunol.182.3.1740 19155523

[12] Eming R Hennerici T Bäcklund J Feliciani C Visconti KC Willenborg S. Pathogenic IgG Antibodies Against Desmoglein 3 in Pemphigus Vulgaris are Regulated by HLA-DRB1*04:02-Restricted T Cells. J Immunol (Baltimore Md 1950) (2014) 193:4391–9. doi: 10.4049/jimmunol.1401081 25252957

